# 2450-MHz microwave ablation of liver metastases under 3.0 T wide-bore magnetic resonance guidance: a pilot study

**DOI:** 10.1038/s41598-022-16989-4

**Published:** 2022-07-25

**Authors:** Kaihao Xu, Zhaonan Li, Yiming Liu, Zaoqu Liu, Chaoyan Wang, Dechao Jiao, Xinwei Han

**Affiliations:** 1 Department of Interventional Radiology, The First Affiliated Hospital of Zhengzhou University, No. 1 Jianshe East Road, Zhengzhou, 450052 Henan China; 2 Department of MRI, The First Affiliated Hospital of Zhengzhou University, Zhengzhou, Henan China

**Keywords:** Gastrointestinal cancer, Liver cancer

## Abstract

To investigate the feasibility and effectiveness of 3.0 T wide-bore magnetic resonance (MR)-guided microwave ablation (MA) of liver metastases (LM). From October 2018 to May 2020, 39 patients with 63 LM were treated with 3.0 T wide-bore MR-guided 2450-MHz MA therapy. The procedure parameters, technical success, complications, biochemical index changes, local tumor response, local tumor progression (LTP), 12-month disease-free survival (DFS) and 12-month overall survival (OS) were recorded and analyzed. The mean tumor maximum diameter and total procedure time were 3.0 cm and 55.2 min, respectively. Technical success was 100%, but 5 cases (12.8%) had grade-1 complications. Alanine transaminase, aspartate transaminase and total bilirubin showed a slight transient increase on day 3 (P < 0.05) and returned to normal by day 30 (P > 0.05). The complete ablation rates for ≤ 2.5 and > 2.5 cm lesions were 100% and 92.5%, respectively. During the median follow-up of 12.0 months, the LTP rate was 4.8% (3/63), and the 12-month DFS and OS rates were 61.3% and 92.2%, respectively. 3.0 T wide-bore MR-guided MA for LM is a safe and effective approach, especially for small LM.

## Introduction

Liver metastases (LM) from primary tumors of the digestive tract are very common; their management often calls for multidisciplinary expertise and follows different pathways, also based on the primary tumor type. For example, colorectal cancer, pancreatic endocrine tumors, and gastrointestinal stromal tumors are often localized and suitable for local treatment, such as resection or ablation, while pancreatic, gastric, and esophageal cancer often spread widely, requiring local treatment in combination with systematic treatment^1^. Unfortunately, only 20% of patients can undergo surgical resection^2^. To improve the local efficacy and reduce systemic side effects, minimally invasive thermal ablation of the LM has been widely used in our clinical practice. The imaging technology used for guiding percutaneous thermal ablation treatments must ensure adequate visualization of the target tumor and of the ablation probe, as well as proper intraprocedural monitoring of treatment progression^3^. LM are typically ablated under ultrasound (US) and/or computed tomography (CT) guidance; however, US may not clearly display small lesions, especially when located near the diaphragm, intestine, or hilum, whereas CT guidance involves the use of ionizing radiation and does not always allow for accurate intraprocedural appraisals of the ablation area^4,5^. Magnetic resonance (MR) has the advantages of high tissue resolution, no ionizing radiation, any multiplanar reconstruction, and accurate ablation area evaluation^6^. Therefore, MR could be an ideal guidance tool for tumor ablation. MR can be divided into two types: open and closed MR. The former provides high patient comfort but low magnetic field intensity (0.2–1.0 T) affecting image quality; the latter provides high magnetic field intensity (1.5–3.0 T) with stability and good image quality but limited interventional operation space because of its closed design and the need for repeated pulling of the examination table in and out of the cylindrical magnets during targeting maneuvers. Moreover, all interventional materials (such as generators, applicators and coaxial cables) must be MR-compatible, which still suffers from limited commercial availability, and 3.0 T closed MR is almost exclusively used for diagnostic purposes; therefore, interventional MR-guided ablation-based therapy is still in its infancy^7^. In this study, we conducted a preliminary evaluation of the feasibility and efficacy of 3.0 T wide-bore MR-guided MA for LM to explore the advantages and disadvantages of this strategy.

## Material and methods

### Patients

The institutional review board of the First Affiliated Hospital of Zhengzhou approved this retrospective study (ethical number: 2018-KY-038), and the protocol used adhered to the tenets of the Helsinki Declaration. All patients provided their informed consent before participation. From October 2018 to May 2020, 39 patients (22 men and 17 women; mean age, 58.1 ± 7.9 years; age range, 43–76 years) with 63 LM were treated with 3.0 T wide-bore MR-guided MA therapy. In all, 22 patients (56.4%) had a single lesion, 10 (25.7%) had two lesions, and the remaining 7 (17.9%) had three lesions. Two interventional radiologists (JDC and WCY with 11 and 5 years of MA experience, respectively) recorded all data, such as the maximum lesion diameter, on the pretreatment images. More detailed information is presented in Table 1. The inclusion criteria were (1) age range: 18–75 years old; (2) LM are still progressing after systematic treatments; (3) LM confirmed by pathological or imaging diagnosis; (4) liver lesions ≤ 3 and single tumor diameter ≤ 5 cm; (5) Child–Pugh A or B; (6) no portal vein thrombus and extrahepatic metastases; (8) Eastern Cooperative Oncology Group (ECOG) score ≤ 2; (9) platelet count > 40 × 10^9^/L and PT ≤ 25 s. The exclusion criteria were (1) intractable ascites; (2) lesion number > 3 or diameter > 5 cm; (3) ECOG score > 2; (4) expected survival time ≤ 3 months; and (4) claustrophobia or MRI-related contraindications. Detailed information is listed in Table 1.

**Table 1:** Patient characteristics.

Data collection	Value (number, mean ± SD, range or %)
Number of patients	39
Male/female	22/17
Age (years)	58.1 ± 7.9 (43.0–76.0)
Primary diagnosis (CC/GC/EC/PC)	17/14/5/3
Primary treatment (S/C/I)	39/33/11
Total lesions (1/2/3)	22/10/7
**Location of lesions**	63
Segment VIII	19
Segment VI	15
Segment IV	11
Segment III	9
Segment VII	5
Segment V	4
**Mean max. diameter (cm)**	3.0 ± 1.0 (0.8–4.6)
≤ 2.5 cm (n = 23)	1.9 ± 0.4 (0.5–2.5)
> 2.5 cm (n = 40)	3.6 ± 0.6 (2.6–4.6)
ECOG score (0/1/2)	20/13/6
Body mass index (kg/m^2^)	24.2 ± 4.1 (17.2–32.6)

SD: standard deviation; S: surgery; C: chemotherapy; I: Immunotherapy; CC: colorectal cancer; GC: gastric cancer; EC: esophageal cancer; PC: pancreatic cancer.

### Instruments

The microwave delivery system used was the ECO-100E MR-compatible microwave apparatus (ECO Medical Instrument Co., Ltd. Nanjing, China) with a power supply voltage of 220 V, frequency of 50 Hz, microwave power of 2450 MHz, and output power of 0–100 W. The MA applicator (ECO-100AI13) was a water-cooled-shaft antenna (1.8 × 150 mm) with a 1.5-cm active tip, and the antenna was cooled to 10–14 °C with a cold-water circulation cooling system (water was circulated by a flow pump at 40–60 mL/min). The MR-compatible extension coaxial cable measured 4.0 m. A Magnetom Verio 3.0 T scanner with an aperture of 70 cm (Siemens, Germany) with its own 6-channel torso body array coil (3 T body Matrix) was used; it has a rectangular square hole that is convenient for interventional procedures. The most commonly used scanning sequence was the T1WI gradient echo volume interpolation body part inspection sequence (T1 Vive, 16 s) and T2WI single-shot half Fourier haste breath holding sequence (T2 Haste, 16 s). Other sequences are listed in Table 2. The body surface was marked with MK-MR-6 (Medical mark Medical Equipment Co., Ltd., Jinan, China).

**Table 2:** Sequence details.

Section	Sequence	TE (ms)	TR (ms)	Slice thickness (mm)	Matrix	Flip angle	Band width (Hz/pixel)
Transverse section	T1 Vive	1.93	4.56	3.3	216 × 288	9.0	400
Transverse section	T2 Haste	106	1000	4.5	137 × 256	180	781
Transverse section	Diffusion	83	7100	5.0	192 × 144	90	1670
Coronal section	T1 Vive	2.46	6.11	3.0	179 × 256	9.0	410
Sagittal	T2 Haste	106	1000	4.0	137 × 256	180	781

### Procedure

The patient fasted for 4 h before the procedure, and venous access was established prior to starting it. The patient’s position was determined according to the preoperative puncture plan on CT/MR. Routine electrocardiogram and oxygen saturation monitoring (Invivo, Orlando, USA) and the ECO-100E MR-compatible MA system were placed on the magnetic-compatible operating table. Dexmedetomidine (0.5 µg/kg) and tropisetron (8 mg) were injected intravenously 10 min before the procedure, and digoxin (5 mg) was injected intravenously 5 min before ablation. After body surface marking with a cod liver oil capsule matrix, a standard MR protocol was performed to locate the hepatic lesions. Diffusion-weighted imaging (DWI) or contrast-enhanced T1 was needed in cases of no clear tumor visualization. Detailed MR sequences are listed in Table 2. After the puncture point was sterilized, 2% lidocaine was used for local anesthetic induction. Then, a skin incision was made using a knife, following which an MA applicator was inserted into the liver, and T1 and T2 scans were carried out several times to confirm the applicator’s position. After satisfactory positioning and placement of the microwave antenna, the ablation parameters were selected according to the lesion size and the manufacturer’s suggestions. The satisfactory microwave antenna position refers to the complete necrosis of the LM and its surrounding area of 0.5–1.0 cm after ablation, and the single cycle microwave ablation parameters are 55–70 W/6–10 min, and repeated multiple MR scans to ensure complete tumor ablation. Cold-water circulation and microwave generator systems were connected to the power supply and carried out the treatment. The applicator was not immediately withdrawn after ablation. Another round of T1WI and T2WI scanning was conducted. If MR showed that the ablation area did not cover the LM and its surrounding area was approximately 0.5–1 cm, the applicator was repositioned, and multiple overlapping ablations were needed. After the operation, the applicator was pulled out, intravenous anesthesia was discontinued, and the patient was returned to the ward, where they were treated with hepatoprotective, acid suppressive, and antiemetic treatments.

### Definition

We recorded all data, such as primary diagnosis, distance from skin to tumor, ablation power, ablation time, total procedure time, MR acquisitions, duration of hospital stay, disease-free survival (DFS) and overall survival (OS). DFS refers to the time span from the beginning of LM ablation to disease recurrence or (for any reason) death, while OS refers to the time span from the beginning of LM ablation to death. A successful puncture should be that the microwave applicator’s position consisted of the operator's ablation strategy, and the LM can be completely ablated within 2 times. The puncture performance was assessed using a 5-point score: (1) unsuccessful MA applicator puncture; (2) successful MA applicator puncture but more than 8 readjustments; (3) successful MA applicator puncture but more than 5–7 readjustments; (4) successful MA applicator puncture but more than 2–4 readjustments; (5) successful MA applicator placement for the first puncture.

Ablation-related complications were evaluated according to the criteria of the Common Terminology Criteria for Adverse Events (CTCAE) version 4.03. Complete ablation (CA) refers to the tumor area showing complete devascularization (at enhanced CT or MR) after ablation, whereas incomplete ablation (ICA) refers to the tumor area showing incomplete devascularization. Local progression was defined as the recurrence of local abnormally enhanced nodules after 4 months of complete tumor ablation.

### Follow-up

Tests for blood counts and liver and renal function were evaluated at 0, 3, and 30 days and compared with each other. 0 day was defined as 3 days before liver tumors ablation in our study. Upper abdomen dynamic-enhanced 3.0-T MR or 64-row CT scans were performed to evaluate the ablation results at 1 month after the procedure. Imaging follow-up was performed every 2 months after complete ablation.

### Statistical analysis

All continuous data are expressed as the mean ± standard deviation or range. The paired t test was performed on continuous data before and after the procedure. The Kaplan–Meier method was used to examine overall survival. P < 0.05 was considered to indicate statistical significance (SPSS software, IBM Corp, Armonk, New York).

## Results

### Patient characteristics and MR manifestations

**Figure 1:**
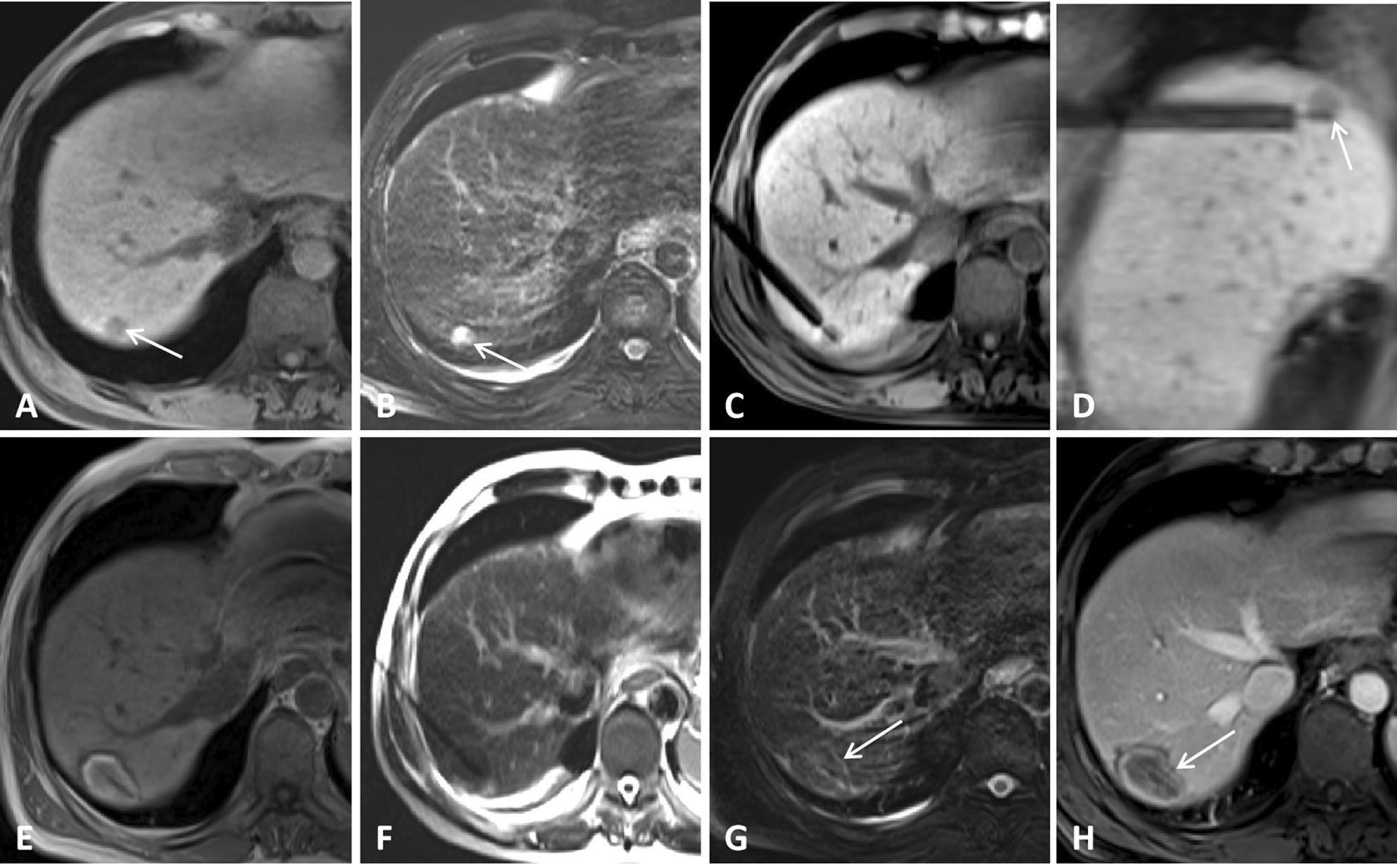
A 62-year-old man with gastric carcinoma and liver metastasis at segment 7. (**A**,**B**) Pretreatment MR showed a small lesion (diameter = 0.9 cm) on T1WI (**A**) and T2WI (**B**); (**C**,**D**) Lesion was punctured by a microwave applicator on cross section (**C**) and sagittal sections (**D**); (**E**) T1WI showed that the ablation focus was ellipsoidal with a high signal, and the central low-density area was the primary tumor; (**F**,**G**) T2WI showed that the high signal of the original tumor disappeared in the nonfat-suppression phase (**F**) and fat-suppression phases (**G**); (**H**) Enhanced MR showed that the focus was completely ablated at the 1-month evaluation (white arrow).

**Figure 2:**
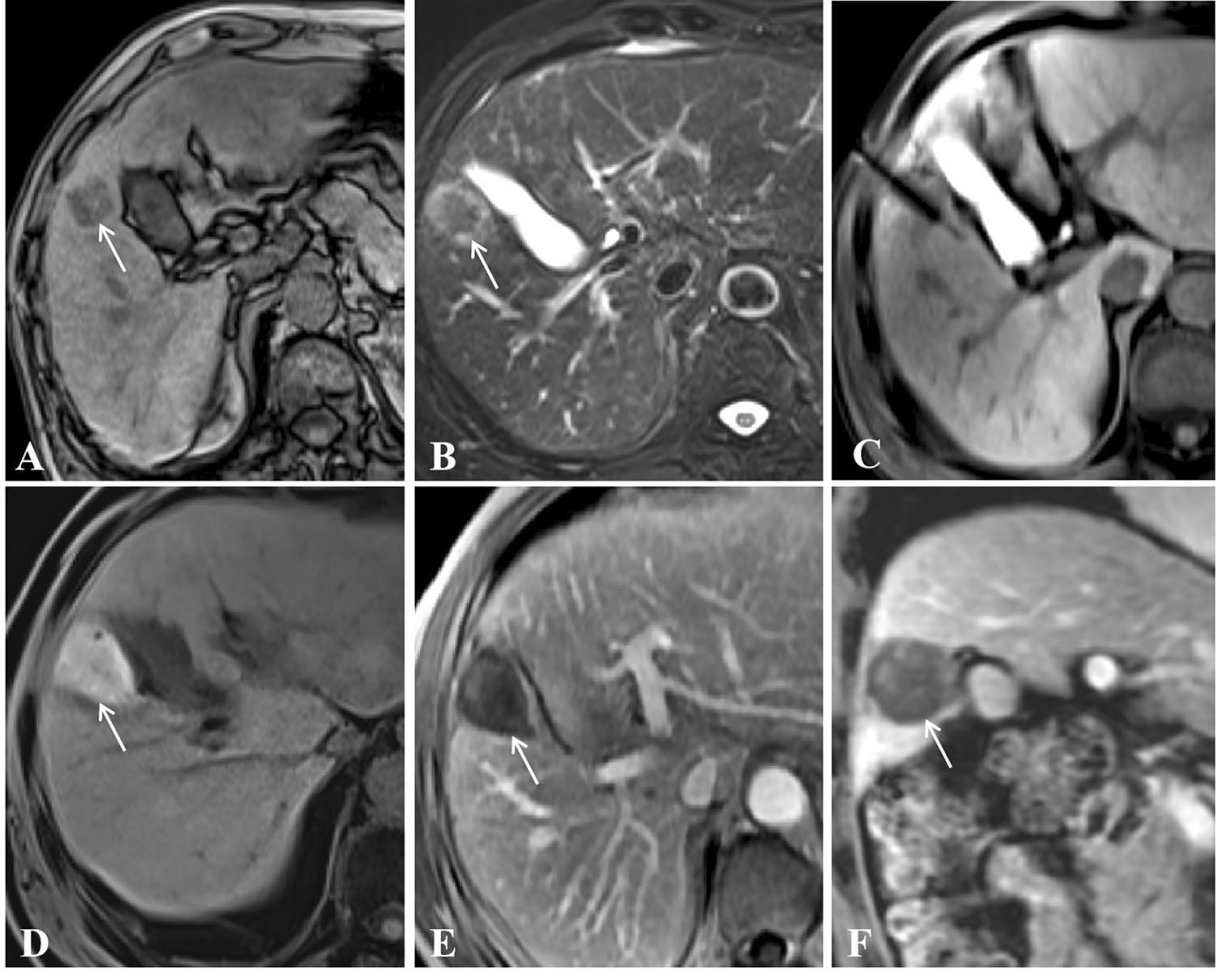
A 56-year-old man with rectal carcinoma and liver metastasis close to the gallbladder at segment 5. (**A**,**B**) Pretreatment MR showed a small lesion (diameter = 2.2 cm) on T1WI (**A**) and T2WI (**B**); (**C**) Lesion was punctured by a microwave applicator on cross section; (**D**) T1WI showed that the ablation focus was ellipsoidal with high signal; (**E**,**F**) Enhanced MR showed that the focus was completely ablated at the 1-month evaluation in cross and sagittal sections (white arrow).

**Figure 3:**
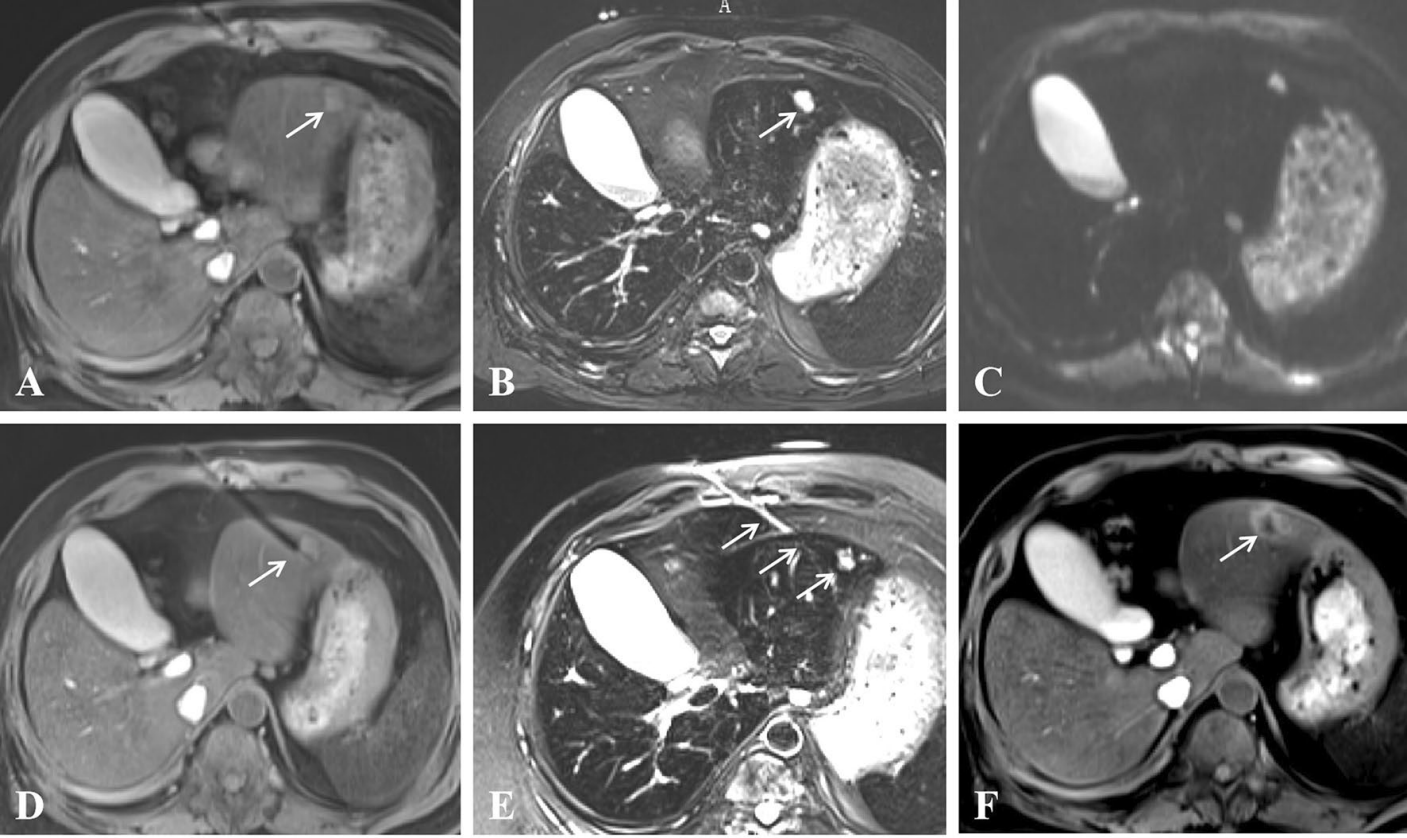
A 66-year-old man with liver metastasis at segment 3 (primary diagnosis was rectal carcinoma and underwent surgical resection). (**A**–**C**) Pretreatment MR showed a small lesion (diameter = 1.3 cm) on T1WI (**A**), T2WI (**B**) and DWI (**C**); (**D**,**E**) Lesion was punctured by a microwave applicator on T1WI (**D**) and T2WI (**E**), and high signal exudation associated with puncture directed by white arrows on Figure (**E**); (**F**) T1WI showed that the ablation focus was ellipsoidal with a high signal, which means complete ablation.

**Figure 4:**
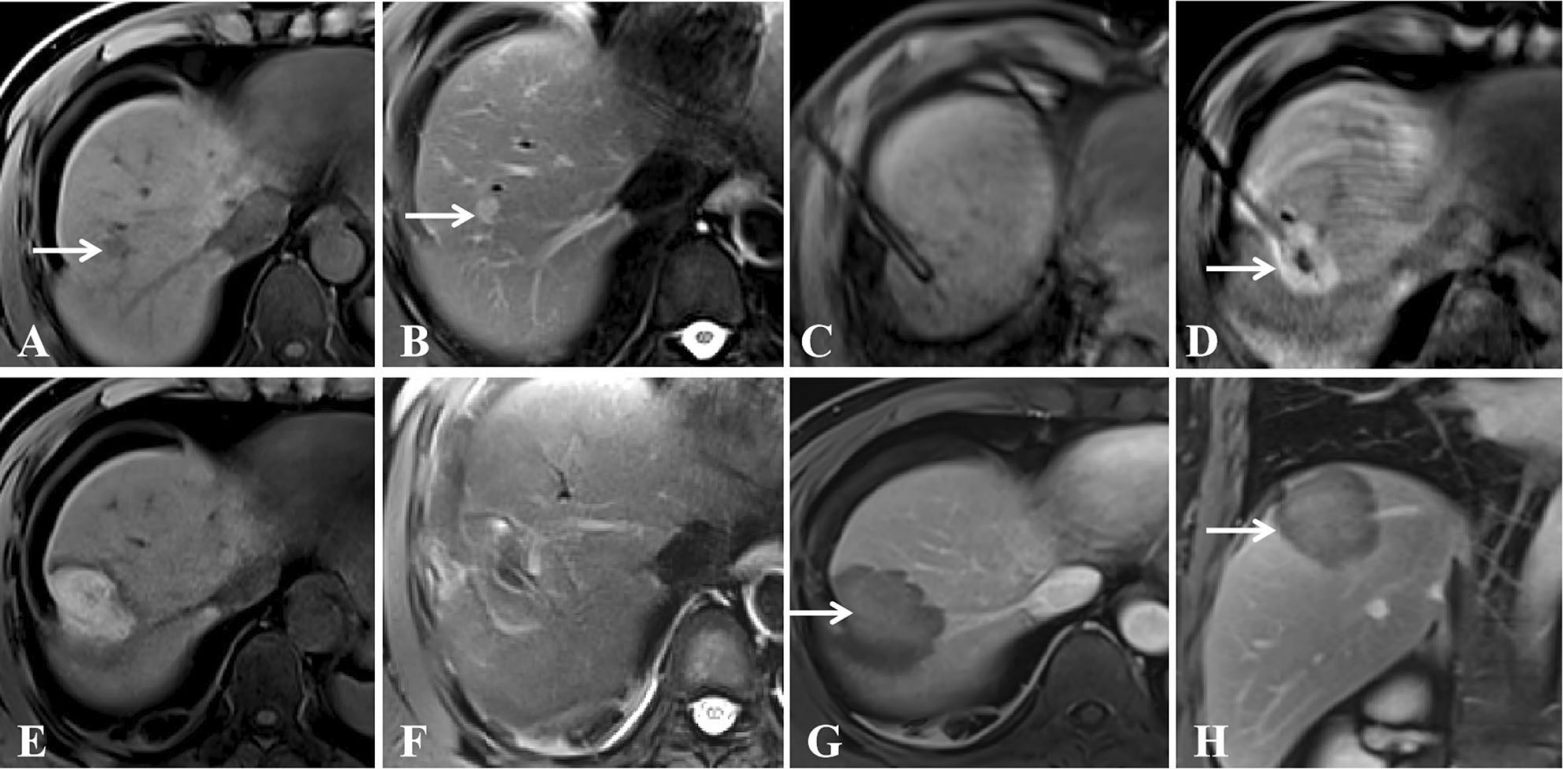
A 54-year-old woman with liver metastasis at segment 8 (primary diagnosis was pancreatic carcinoma). (**A**,**B**) Pretreatment MR showed a small lesion (diameter = 1.1 cm) on T1WI (**A**) and T2WI (**B**); (**C**) The lesion was punctured by a microwave applicator on T1WI; (**D**) The high signal covered the primary tumor after the first circle of microwave ablation (white arrow); (**E**,**F**) Posttreatment MR showed that the ablation zone had a high signal on T1WI (**E**) and a low signal on T2WI (**F**); (**G**,**H**) Enhanced MR showed that the focus was completely ablated at the 1-month evaluation in cross (**G**) and sagittal sections (**H**) (white arrow).

The mean distance from the skin to the liver lesion was 10.7 cm (range, 7.4–14.6), and the mean MR scan acquisition for a single lesion was 29.8 times (range: 16–52). Applicator punctures were successful in all cases. The mean ablation power (measured at the generator output port) and time taken for ablation of a single lesion were 64.8 ± 4.6 W and 15.4 ± 7.0 min, respectively. The mean score of puncture performance was 4.0 (range, 2–5), and 19 (30.2%) lesions were punctured once. More detailed information is provided in Table 3. For MRI manifestations, the MA needle showed a low signal during the ablation, and the applicator tip image was acceptable. If necessary, scanning along the needle path was performed, which clearly showed the depth of the puncture needle. On T1WI, the ablation focus appears as a high-signal area with clear boundaries, and the relatively low-signal area in the center is the initial tumor. The high-signal area became larger and clearer as time progressed. Scanning is recommended 5–8 min after ablation, as the high-signal ablated area can be better visualized. The T2WI ablation zone showed a low signal, and a high-signal loop was seen around it (Figs. 1, 2, 3, 4).

**Table 3:** Intra- and post-operative details.

Data	Value (number, mean ± SD, range or %)
Technical success	110 (100)
Distance from skin to target (cm)	10.7 ± 2.2 (7.4–14.6)
Mean score for puncture performance	4.0 ± 0.8 (2–5)
Tumor number of score 5	19 (30.2)
Tumor number of score 4	28 (44.4)
Tumor number of score 3	13 (20.6)
Tumor number of score 2	3 (4.8)
Mean ablation power	64.8 ± 4.6 (55–70)
Mean ablation time	15.4 ± 7.0 (6–32)
Total procedure time	55.2 ± 10.4(46.0–85.5)
Number of MR acquisitions for single lesion	29.8 ± 3.8 (16–52)
Mean hospital stay (days)	6.1 ± 2.2 (2–11)
Minor complications (Grade-1)	5 (12.8)
**Tumor response at 2-month (CR/PR)**	
≤ 2.5 cm	23/0
> 2.5 cm	37/3
Followup period (month)	12.0 ± 3.8 (4–18.5)
Local progression	3 (4.8%)
Followup treatment (chemotherapy/irradiation /immunotherapy)	24/6/6
Overall survival rate (6/12/18-month survival)	100/92.2/76.4

CA: complete ablation.

### Safety and complications

**Figure 5:**
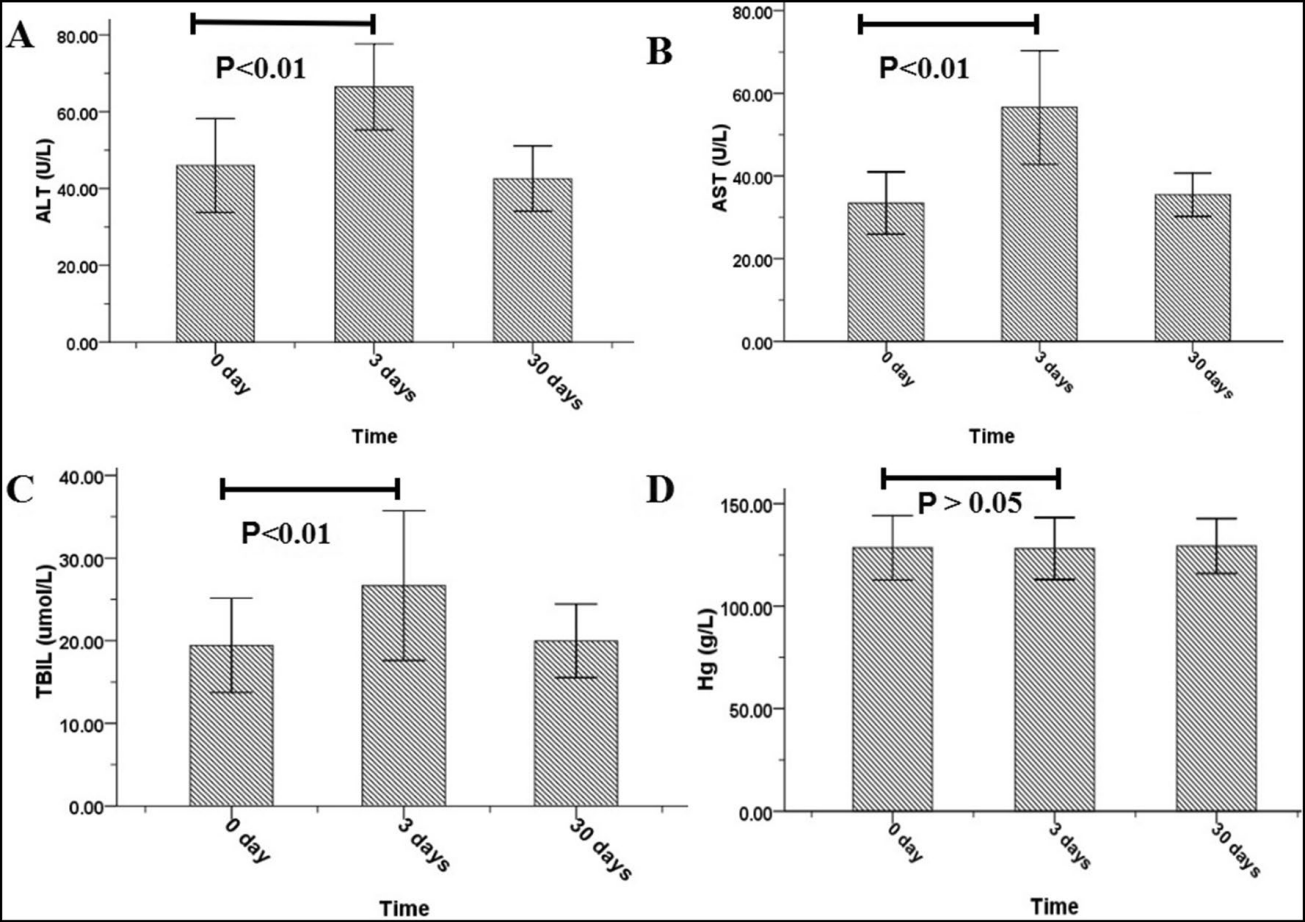
Changes in liver function and hemoglobin were observed at 0, 3 and 30 days.

The results of blood counts (white blood cell [WBC], hemoglobin [Hg], platelet [PLT]) and levels of creatinine (Cr); blood urea nitrogen (BUN), alanine transaminase (ALT), aspartate transaminase (AST), and total bilirubin (TBIL) on day 0 (preablation day), day 3, and day 30 are summarized in Table 4. ALT, AST, and TBIL showed transient increases on day 3 (P < 0.05) but returned to baseline on day 30. WBC, Hg, PLT, Cr, and BUN showed no significant changes among day 0, day 3, and day 30 (P > 0.05) (Fig. 5).

**Table 4:** Biochemical indexes at 0- (pre-ablation) and 3-, and 30-days post-ablation.

Parameter	0-day	3-day followup	30-day followup	P1 value	P2 value
WBC (× 10^9^/L)	6.4 ± 1.4	6.0 ± 1.6	5.9 ± 1.4	0.07	0.26
PLT (× 10^11^/L)	205.0 ± 43.6	203.8 ± 42.1	205.6 ± 42.9	0.29	0.73
Hg (g/L)	128.5 ± 15.7	128.1 ± 15.1	129.4 ± 13.4	0.17	0.35
ALT (U/L)	43.2 ± 9.9	63.9 ± 12.6	40.5 ± 5.3	0.00	0.08
AST (U/L)	36.2 ± 5.3	56.6 ± 13.8	35.5 ± 5.2	0.00	0.42
TBIL (µmol/L)	19.4 ± 5.7	26.7 ± 9.0	20.0 ± 4.5	0.00	0.53
Creatinine (µmol/L)	64.7 ± 17.6	68.2 ± 15.0	66.2 ± 15.2	0.15	0.66
UN (mmol/L)	4.9 ± 1.1	5.2 ± 1.5	5.1 ± 1.2	0.12	0.77

P1 and P2 values for 0-day vs. 3-day and 0-day vs. 30-day parameters, respectively.

WBC: white blood cells; PLT: platelets; Hg: hemoglobin; ALT: alanine transaminase; AST: aspartate transaminase; TBIL; total bilirubin; UN: Urea nitrogen.

During the operation, five patients (12.8%) had moderate pain due to the lesion near the liver capsule. The pain could be tolerated after intravenous injection of diazepam. Five patients (12.8%) with S8 lesions had postrespiratory pain after ablation, and CT showed localized pleural effusion (grade 1 adverse event). The symptoms were relieved after symptomatic treatment. Eleven patients (28.2%) showed transient heat absorption (37.6–38.7 °C); they were prescribed antipyretic medication and instructed to drink plenty of water, after which their body temperature returned to normal within 2–4 days. No serious complications, such as liver abscess, bile lake, hemorrhage, diaphragmatic perforation, or severe jaundice, were observed.

### Local response

The complete ablation rates for lesions measuring  ≤ 3 and > 3 cm were 100% and 92.5% at the 1-month evaluation, respectively. Two lesions (diameter = 4.2 cm, S5; diameter = 4.5 cm, S8) had a local residual tumor close to the pleural edge, and another lesion (diameter = 4.6 cm, S7) had a residual tumor close to the portal vein. Both of these lesions required secondary MA to control the local residual tumor.

### DFS and OS

**Figure 6:**
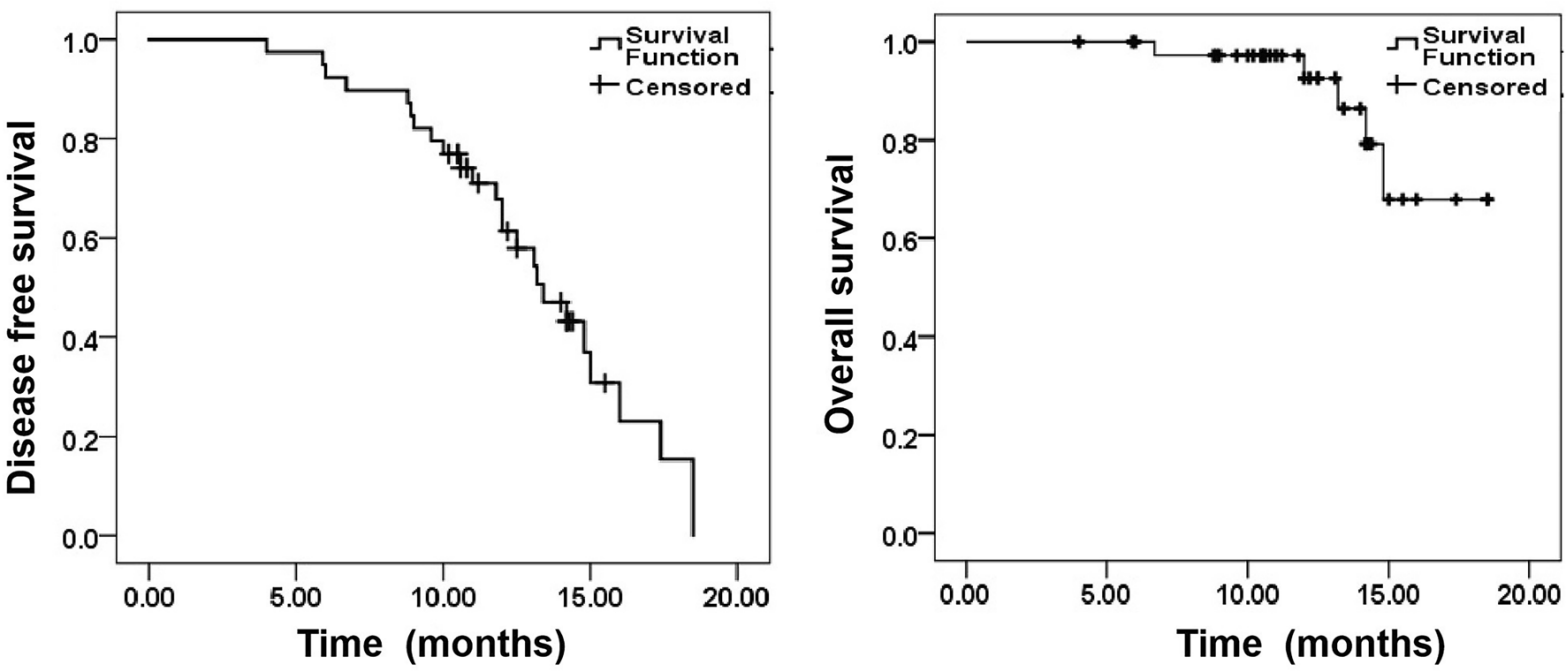
The disease-free survival and overall survival of 39 cases.

Five patients died during the mean follow-up of 12.0 months (range: 4–18.5), two of them (1 patient with low differentiation colorectal cancer and 1 patient with low differentiation gastric cancer) died due to disease progression resulting from multiple organ failure. Two patients died of sudden myocardial infarction, and one patient died of intractable gastrointestinal bleeding (unknown cause). Local tumor progression occurred in 3 cases (4.8%). Two lesions were located under the diaphragm (primary diameter = 4.5 cm and 4.2 cm), and one lesion was located close to the liver capsule (primary diameter = 4.0 cm), among whom 2 cases underwent ^125^I seed implantation and 1 case underwent MA again to control the local tumor. Extrahepatic metastasis occurred in 17 cases. The 6-, 12- and 18-month DFS rates were 92.3%, 61.3% and 15.4%, respectively. The 6-, 12- and 18-month OS rates were 100, 92.2%, and 67.9%, respectively (Fig. 6).

## Discussion

Due to the anatomical and histological characteristics of the dual hepatic blood supply, the liver is a common metastatization site for all malignant tumors of the digestive tract, such as colorectal cancer -leading to hepatic metastases in over 50% of the cases-, gastric cancer, pancreatic cancer, etc. At present, the best strategy for LM is surgical resection. However, not all patients are eligible to undergo resection owing to poor liver function, multiple lesions, unique lesion location, advanced age, contraindication of general anesthesia, and subjective rejection or refusal to be operated upon^8^. During the past 30 years, image-guided ablation techniques such as MA, radiofrequency ablation (RFA) and cryo-ablation have shown rapid development to become useful local treatment protocols for LM^9,10^. An increasing number of basic and clinical studies have shown that MA can generate, deposit, and transmit heat energy more quickly than conventional RFA, which means that MA may increase ablation efficacy and reduce the “heat-sink” effect when the tumor is close to the large blood vessel^11,12^. In theory, the preferable image-guidance modality should provide convenient, fast, accurate, and efficient guidance, along with precise and instant evaluation of the lesion^13^. At present, mainstream ablation-guidance modalities such as CT or ultrasound have their own advantages and disadvantages. Ultrasound imaging does not expose patients to ionizing radiation and it provides real-time monitoring with any-angle imaging function, but it is limited by artifacts induced by gas formation during thermal treatments and by the hindrance of bone tissues to elastic waves propagation^14^. While contrast-enhanced ultrasound can better display the characteristics of tissue enhancement after ablation, the bubbles produced by local high temperature after ablation can lead to high echogenicity, thereby causing overestimation of the ablated area^15^. Multislice spiral CT has the advantages of fast scanning and wide adaptability, but it involves exposure to ionizing radiation and shows no accurate assessment of the ablation boundary without immediate enhanced CT^16^.

This pilot study showed that the technical success was 100%, and the CA rates for lesions measuring ≤ 2.5 and > 2.5 cm were 100% and 92.5%, respectively, without severe complications. MR possesses many advantages, such as very high soft-tissue resolution, multiparameter imaging, and free ionizing radiation exposure. There are very few clinical studies describing MR-guided MA, as most of them are carried out under 0.5–1.0 T open low-field magnets^15,17–22^ (Table 5). Theoretically, soft tissue resolution is higher in field strengths > 1.5 T than in open low field strength, which means more technical advantages in the detection, monitoring and evaluation of LM and MA results. In 2000, Chen et al.^15^ reported five patients with prostate tumors who underwent 1.5 T MR-guided MA. Although the reported technical success was 100%, this method did not have wide clinical application owing to technical limitations such as the microwave generator needing to be arranged outside the MR room and the long-distance coaxial cable producing additional noise. The microwave ablation machine used in our study can be placed within the 5 Gauss line, and there is no obvious interference on the images. We believe that it can be more widely used in the future.

**Table 5:** Clinical studies published on MR-guided Microwave ablation.

Authors	Year	Pts number	MR-type	Study design	Study objective	Tumor type	Microwave equipment	Results
Chen et al.^16^	2000	5 pts	1.5 T whole-body MR(GE)	Rs	Safety and feasibility	Prostate cancer	Urowave®, Dornier, 915 MHz, Germany	Technical success100%
Morikawa et al.^17^	2002	30 pts	0.5 T open MR (GE)	Rs	Safety and feasibility	Liver metastases	Microtaze®, OT-110 M, 2450 MHz, Japan	Satisfactory results
Morikawa et al.^18^	2004	33 pts	0.5 T open MR (GE)	Rs	Respiratory triggering for ablation	Liver tumor	Microtaze®, OT-110 M, 2450 MHz, Japan	Feasibility of respiratory triggering for ablation under general anesthesia
Abe e t al.^19^	2005	8 pts	0.5 T open MR (Philips)	Rs	Safety and feasibility	liver metastases	Microtaze®, OT-110 M, 2450 MHz, Japan	Technical success 100%
Murakami et al.^20^	2015	6 pts	0.5 T open MR (GE)	Rs	Feasibility for MR guided laparoscopic ablation	Liver tumors	Microtaze®, HSD20M, 2450 MHz, Japan	Effective treatment for tumor ablation avoiding adjacent organs
Hoffmann et al.^21^	2016	11pts	1.5 T wide-bore MR (Siemens)	Ps	Safety an effectiveness	Liver tumors	Medwaves AvecireTM, 928 MHz, USA	Near real-time MR guidance for ablation
Lin et al.^22^	2019	35 pts	1.5 T whole body MR (GE)	Rs	Safety and feasibility	Liver tumors	Vision®, MTC-3CA-II, 2450 MHz, China	Technical efficacy 100%
Current study	2021	39 pts	3.0 T wide-bore MR (Siements)	Ps	Safety and feasibility	liver metastases	ECO-100E®, 2450 MHz, China	Technical success 100%

Pts: patients; RS: retrospective study; Ps: perspective study.

The difference between wide-bore MR (bore diameter: 75 cm) and conventional closed MR (bore diameter: 65 cm) lies in the following three points: (1) the cross-sectional area of the former is increased by 33.1% compared with the latter; (2) the increased space provides more convenience for puncture; (3) the increased space reduces claustrophobia and improves the comfort of patients during MR guidance even for obese cases; and (4) the disadvantage is that the wide-bore MR cost will also increase. Liver function showed a transient increase and returned to baseline by day 30 post MA, which meant that the tissue damage was temporary. The results are expected because we used the same ablation process as previous CT or US guidance. Furthermore, the MR-based unique signal change can be used as an objective, reliable, and rapid assessment tool given the high signal on T1WI, low signal on T2WI, and sharp contrast with the primary tumors in the ablation zone. The 3.0 T high field strength resulted in high soft-tissue resolution, thus capturing the signal changes of the boundary after ablation for instant precise evaluation. Such typical changes are mainly due to the characteristics of water decrease in tissues. Sheng et al.^23^ and Lee et al.^24^ both agreed that the “target sign” unenhanced T1WI scan was enough to evaluate the ablation extent and identify the residual tumor. On the other hand, CT can only distinguish between high and low density. Ablation leads to low tissue density, while ablation edge tissue hemorrhage and edema lead to a local density increase that in turn affects the accurate evaluation of the edge^25^. The formation of air bubbles during ultrasound-guided ablation results in a blurred boundary. Thus, MR-guided ablation plays a very important role in the ablation of very small lesions and lesions at specific locations^26,27^. It has been reported that poor display of the microwave applicator tip (length, 1.6 cm) on T1WI may be related to the ceramic structure of the tip, especially for oblique noncoplanar puncture, but the depth and shape of the applicator tip can be observed better after scanning along the long axis of the microwave applicator^28^. Two lesions close to the pleural and portal vein had residuals after the first MA, which required another MA again, which means that special sites or “heat-sink” have the same impact on MR-guided ablation as other tools. Due to the complex tumor composition in this study, the 18-month OS (67.9%) may not be accurate. In the future, it is necessary to increase the sample size, extend the observation time and carry out stratified analysis on tumors to summarize the ablation effect.

There are still some unaddressed disadvantages of MR guidance, such as long scanning time, being unsuitable for patients with poor breath-holding coordination, high cost of MR-compatible applicator adding to the treatment costs, a closed magnet system that offers very limited procedural convenience, and being contraindicated in patients with artificial pacemaker, metal implants, and claustrophobia. This pilot study is limited by its retrospective design, the short follow-up duration and the lack of a control group. Further randomized controlled trials comparing wide-bore 3.0 T MRI guided MA interventions to US-guided and/or CT-guided interventions are warranted to investigate and quantify the theoretical advantages offered by MR guidance, especially in the treatment of tumors less than 15 mm in size.

This pilot study showed that the 3.0 T wide-bore MR-guided MA for LM is safe and effective. We believe that with adequate medical resource reallocation to make wide-bore MR and MR-compatible microwave materials more accessible, interventional MR has strong potential for wide and successful clinical application.
